# CDI Systems Are Stably Maintained by a Cell-Contact Mediated Surveillance Mechanism

**DOI:** 10.1371/journal.pgen.1006145

**Published:** 2016-06-29

**Authors:** Zachary C. Ruhe, Josephine Y. Nguyen, Annette J. Chen, Nicole Y. Leung, Christopher S. Hayes, David A. Low

**Affiliations:** 1 Department of Molecular, Cellular and Developmental Biology, University of California, Santa Barbara, Santa Barbara, California, United States of America; 2 Biomolecular Science and Engineering Program, University of California, Santa Barbara, Santa Barbara, California, United States of America; Uppsala University, SWEDEN

## Abstract

Contact-dependent growth inhibition (CDI) systems are widespread amongst Gram-negative bacteria where they play important roles in inter-cellular competition and biofilm formation. CDI^+^ bacteria use cell-surface CdiA proteins to bind neighboring bacteria and deliver C-terminal toxin domains. CDI^+^ cells also express CdiI immunity proteins that specifically neutralize toxins delivered from adjacent siblings. Genomic analyses indicate that *cdi* loci are commonly found on plasmids and genomic islands, suggesting that these Type 5 secretion systems are spread through horizontal gene transfer. Here, we examine whether CDI toxin and immunity activities serve to stabilize mobile genetic elements using a minimal F plasmid that fails to partition properly during cell division. This F plasmid is lost from *Escherichia coli* populations within 50 cell generations, but is maintained in ~60% of the cells after 100 generations when the plasmid carries the *cdi* gene cluster from *E*. *coli* strain EC93. By contrast, the *ccdAB* "plasmid addiction" module normally found on F exerts only a modest stabilizing effect. *cdi*-dependent plasmid stabilization requires the BamA receptor for CdiA, suggesting that plasmid-free daughter cells are inhibited by siblings that retain the CDI^+^ plasmid. In support of this model, the CDI^+^ F plasmid is lost rapidly from cells that carry an additional *cdiI* immunity gene on a separate plasmid. These results indicate that plasmid stabilization occurs through elimination of non-immune cells arising in the population via plasmid loss. Thus, genetic stabilization reflects a strong selection for immunity to CDI. After long-term passage for more than 300 generations, CDI^+^ plasmids acquire mutations that increase copy number and result in 100% carriage in the population. Together, these results show that CDI stabilizes genetic elements through a toxin-mediated surveillance mechanism in which cells that lose the CDI system are detected and eliminated by their siblings.

## Author Summary

Many bacteria express contact-dependent growth inhibition (CDI) systems, which are used to bind bacteria of the same species together and deliver toxins that block bacterial growth. Sibling cells are protected by a CDI-encoded immunity protein, but unrelated neighbors are inhibited because they lack immunity. Thus, CDI provides a mechanism to identify neighboring cells as "self" or "nonself-". CDI genes are typically found on genomic DNA regions that can move between different bacteria. Here, we find that CDI genes promote the stability of small replicating DNA elements called plasmids, allowing them to be maintained in a population over many generations. If a cell loses the CDI plasmid DNA, then it also loses immunity to toxin and is eliminated by neighboring CDI^+^ cells. We call this a surveillance mechanism because CDI^+^ cells continually deliver toxins to test whether their neighbors are true siblings. Cells lacking the CDI genes are recognized as foreign and are eliminated from the population. This work shows that CDI systems exert a powerful selective pressure and act to stabilize DNA elements with which they are associated.

## Introduction

Many organisms acquire new genetic information through horizontal gene transfer (HGT), which facilitates rapid adaption to new environments. Bacteria, in particular, use HGT extensively to maintain flexible, fluid genomes that support diverse lifestyles. The genes gained through horizontal transfer allow bacteria to exploit new metabolites, acquire antibiotic resistance, and deploy virulence factors during pathogenesis. Some bacteria are able to take up DNA directly from the environment, but many others acquire mobile genetic elements through conjugation, which requires close contact between donor and recipient cells. Genes are also transferred between cells via bacteriophage-mediated transduction [1]. Because foreign DNAs are potentially deleterious, HGT is often limited by anti-viral defense systems like restriction endonucleases and CRISPR-Cas systems, which recognize and destroy foreign DNAs [2]. Nonetheless, HGT occurs between cells and plays a major role in the evolution of bacteria and other organisms [1–4]. Once a mobile genetic element gains access to a new cell, it must replicate either as an episome or integrate into the host genome to be passed on to subsequent generations. Plasmids use several strategies to ensure stable maintenance in bacterial hosts. High-copy plasmids exploit the power of numbers, with only a small statistical chance that a cell will be “cured” of plasmid in each generation. Low-copy plasmids carry DNA sequences that function similarly to centromeres, encoding partitioning proteins that actively segregate plasmid DNA into each daughter during cell division [5, 6]. In addition, low-copy plasmids usually express one or more toxin-antitoxin (TA) systems, which stabilize the element through post-segregational killing [7–9]. TA modules are usually organized as operons with the upstream gene encoding an unstable antitoxin and the downstream gene coding for a stable protein toxin. Type I TA systems use a small RNA to inhibit toxin translation, whereas type II systems produce labile antitoxin proteins that inactivate toxin [10, 11]. Because antitoxin proteins have short half-lives, they must be synthesized continuously to prevent toxin-induced cell death. Thus, daughter cells that fail to receive plasmid can cannot produce antitoxin and eventually succumb to toxin activity. In this manner, TA systems selectively eliminate plasmid-free cells and are sometimes referred to as "plasmid-addiction modules”. This same general mechanism can stabilize chromosomal elements such as prophages and genomic islands [11, 12].

Many Gram-negative bacteria express another class of toxin/antitoxin systems that mediate contact-dependent growth inhibition (CDI) [13–17]. In contrast to type II TA systems, CDI toxins are deployed to inhibit the growth of neighboring bacterial cells. CDI is mediated by the CdiB/CdiA family of two-partner secretion proteins. CdiB is an outer-membrane β-barrel protein that exports large, filamentous CdiA effector proteins to the cell surface. CdiA binds specific receptors on susceptible bacteria [18], then delivers its C-terminal toxin domain (CdiA-CT) to inhibit target-cell growth [19, 20]. CDI^+^ bacteria also exchange toxins with sibling cells, but protect themselves with CdiI immunity proteins, which act as antitoxins to bind CdiA-CT domains and neutralize inhibition activity. CDI systems encode a variety of toxins and immunity proteins, and different strains of the same species commonly possess distinct CdiA-CT/CdiI sequences [14, 21]. Moreover, CDI^+^ cells can bind to both CDI^+^ and CDI^-^ cells via CdiA-CdiA and CdiA-receptor interactions, respectively. Because CdiI-mediated immunity is specific for cognate CdiA-CT toxin, toxin/immunity protein polymorphism suggests that CDI functions in inter-strain competition. In this model, strains deploy distinct toxins to compete for growth niches and environmental resources.

The distribution of *cdi* genes between isolates of a given bacterial species is not necessarily uniform. For example, though every isolate of *Neisseria meningitidis*, *Burkholderia pseudomallei* and *Yersinia pestis* contains at least one *cdi* gene cluster, only a subset of *Escherichia coli* strains carry these systems [17, 22]. These observations indicate that *cdi* genes comprise part of the *E*. *coli* accessory genome and are therefore most likely acquired through HGT. Here, we show that *E*. *coli* CDI systems are commonly encoded on genomic islands and provide evidence that *cdi* toxin/immunity coding regions are subject to frequent HGT. Further, we demonstrate that *cdi* genes from *E*. *coli* EC93 can stabilize a horizontally transferred genetic element. CdiA^EC93^ targets the BamA receptor, delivering a pore-forming CdiA-CT^EC93^ toxin that reduces the proton motive force in non-immune target cells [23]. We engineered a minimal F plasmid that is rapidly lost from *E*. *coli* populations within 50 cell generations. However, this unstable episome is maintained over hundreds of generations when it carries the *cdiBAI* gene cluster from *E*. *coli* EC93. In contrast, the *ccdBA* TA module normally found on the F plasmid has only a modest effect on stability, with the plasmid being lost by 60 generations. We show that *cdi*-dependent genetic stabilization requires inter-cellular toxin exchange and reflects the selection for immunity to CDI-mediated growth inhibition. Thus, *cdi* genes stabilize genetic elements via a surveillance mechanism in which individuals within the population are continually challenged to determine whether they are immune or “self” cells. Individuals that lose the *cdi* locus are rendered non-immune and are targeted for elimination as “non-self” cells.

## Results

### *E*. *coli* CDI systems are encoded on genomic islands

**Fig 1:**
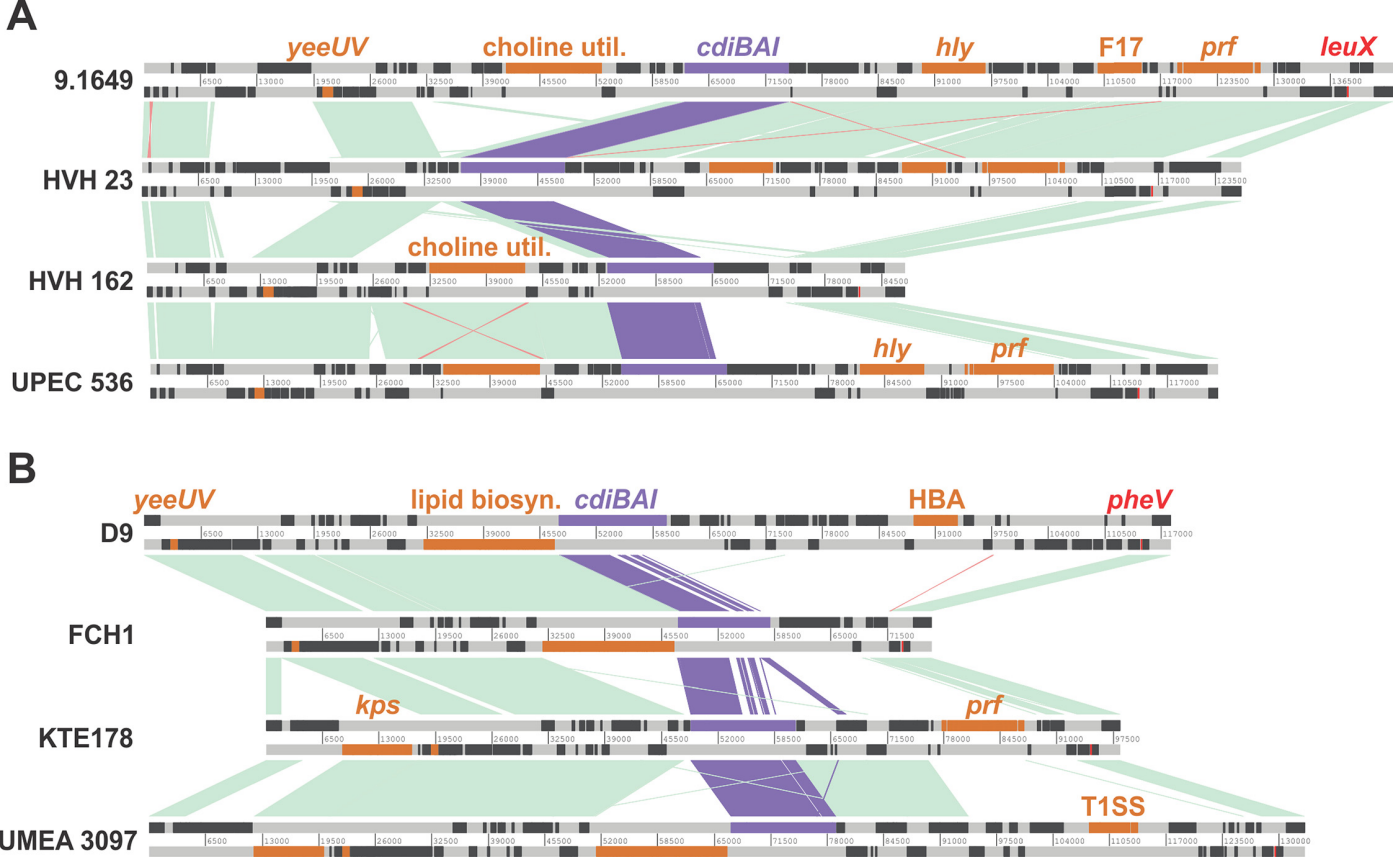
*E*. *coli cdiBAI* gene clusters are located on genomic islands. *E*. *coli* genomic islands harboring *cdiBAI* genes were aligned using the Artemis comparison tool. PAI II islands from 4 different *E*. *coli* strains are shown in panel A. Panel B displays 4 genomic islands inserted at *pheV* in the indicated strains. Homologous CDI DNA sequences are highlighted in blue-violet, homologous non-CDI DNA sequences are shown in light green (direct orientation) and light red (inverted orientation), and genes of interest are shown in orange. Abbreviations: *yeeUV*, toxin-antitoxin module; choline util, genes involved in choline metabolism; *hly*, hemolysin biosynthesis; F17, F17 fimbrial genes; *prf*, P-related fimbriae; lipid biosyn., putative lipid biosynthesis operon; HBA, genes involved in hydroxybenzoate degradation; *kps*, capsular assembly operon; T1SS, Type I secretion system.

To examine the genomic context of *E*. *coli cdi* gene clusters, we used the *cdiA* sequence from *E*. *coli* EC93 to query the *Escherichia* taxon using BLAST. The top 961 hit sequences were then analyzed using JContextExplorer to compare *cdi* gene neighborhoods [24]. This analysis revealed that a large number of *E*. *coli cdi* loci are harbored within genomic islands inserted at the 3´-ends of various tRNA genes including *leuX*, *pheV*, *selC* and *aspV*. The *leuX* integrated islands are related to the previously characterized pathogenicity island 2 (PAI II_536_) from uropathogenic *E*. *coli* 536 (UPEC 536) (Fig 1A) [25]. In addition to the *cdi* locus, PAI II_536_ carries several gene clusters that encode important virulence factors including hemolysin (hly) and fimbriae including Prf and F17 (Fig 1A). Notably, the *leuX* islands are mosaics and contain distinct complements of genes between different *E*. *coli* isolates. For example, the island from *E*. *coli* 9.1649 is closely related to PAI II_536_, but contains an additional F17-like fimbrial operon (Fig 1A). Similarly, the *leuX* island from *E*. *coli* HVH 23 shares most of its genes with PAI II_536_, but lacks the cluster of choline utilization genes found upstream of the *cdi* locus (Fig 1A). The island from *E*. *coli* HVH 162 retains the choline utilization cluster, but lacks the hemolysin and fimbrial virulence genes found downstream of *cdi* in other isolates (Fig 1A). This latter difference is also associated with distinct *cdi* toxin/immunity protein coding sequences in *E*. *coli* HVH 162. The CdiA proteins from UPEC 536, *E*. *coli* 9.1649 and *E*. *coli* HVH 23 all carry the same C-terminal Ntox28 domain (Pfam: PF15605), which has toxic anticodon nuclease activity [14, 26, 27]. CdiA from *E*. *coli* HVH 162 carries a non-specific nuclease toxin domain (Pfam: PF13930) [27]. Thus, additional HGT events shape the gene content of each *leuX* island. The *cdi*-containing islands integrated at *pheV* exhibit similar heterogeneity (Fig 1B). There is also greater diversity in *cdiA-CT/cdiI* coding sequences for these islands. *E*. *coli* D9 encodes the same Ntox28 toxin domain as UPEC 536, but CdiA from *E*. *coli* FCH1 carries a C-terminal DUF4237 (Pfam: PF14021) domain of unknown function. The CdiA-CT domains from *E*. *coli* isolates KTE178 and UMEA 3097 are identical to the toxin from *E*. *coli* NC101 [17, 20]. Comparison of the FCH1 and KTE178 islands shows that the *cdiA-CT/cdiI* sequences from FCH1 have been displaced downstream to form an “orphan” toxin/immunity module (Fig 1B). Such displaced toxin/immunity gene pairs are commonly found downstream of *cdiBAI* clusters in many different bacterial species [28]. In many instances, orphan toxin/immunity pairs form tandem arrays associated with intervening transposase and integrase genes. Together, these observations suggest that *cdi* loci are subject to continual modification through transposition and site-specific recombination.

### *cdi* genes stabilize genetic elements

**Fig 2:**
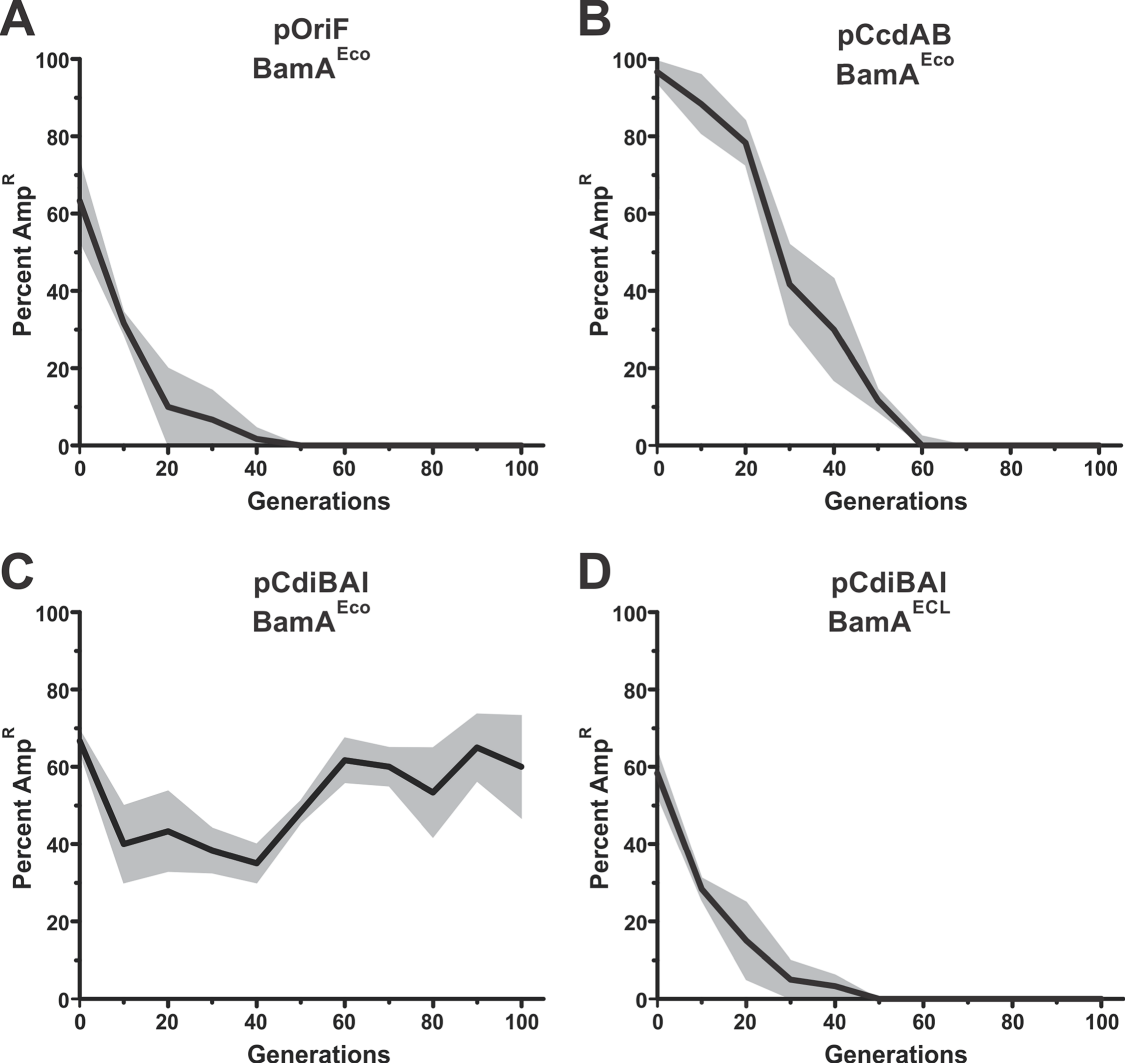
CDI-mediated plasmid stabilization. *E*. *coli* EPI100 populations harboring mini-F plasmid derivatives were passaged daily in broth culture for 100 generations. Randomly selected colonies were screened Amp^R^ after each passage to determine the percentage of cells that retain the plasmid. The average percentage of Amp^R^ colonies is plotted with the standard error indicated by grey shading. **A**) *E*. *coli* EP100 Δ*bamA*::*cat* pZS21-BamA pOri_F_. **B**) *E*. *coli* EP100 Δ*bamA*::*cat* pZS21-BamA pCcdAB. **C**) *E*. *coli* EP100 Δ*bamA*::*cat* pZS21-BamA pCdiBAI. **D**) *E*. *coli* EP100 Δ*bamA*::*cat* pZS21-BamA^ECL^ pCdiBAI.

TA modules stabilize plasmids through a phenomenon called post-segregational killing, in which plasmid-free daughter cells are subject to toxin-mediated killing. Because CDI systems also have toxin (CdiA-CT) and antitoxin (CdiI) activities, we asked whether *cdi* genes have a similar stabilizing effect on genetic elements. Natural genomic islands are stable over long-term passaging [29, 30] and plasmids often harbor several stabilizing elements [31, 32], making it difficult to study the contribution of *cdi* genes in these contexts. Therefore, we generated an unstable ampicillin-resistant (Amp^R^) plasmid, pOri_F_, which uses the F origin to tightly couple plasmid DNA replication to host-cell chromosome replication [33]. Plasmid pOri_F_ does not contain the *sopABC* locus, which encodes the active partitioning system [34]; nor does it carry the *ccdAB* TA module that mediates post-segregational killing [35, 36]. Because pOri_F_ lacks elements that normally stabilize F, this episome allows us to study stabilizing effects in isolation through short-term passaging experiments. When propagated in *recA*^−^host cells, pOri_F_ was lost from populations after about 50 generations (Fig 2A). Introduction of the *ccdAB* TA module increased pOri_F_ carriage, but the plasmid was still lost within 60 generations (Fig 2B). Thus, the *ccdAB* module provides only a modest increase in plasmid stability. We next placed the *cdiBAI*^EC93^ genes from *E*. *coli* EC93 onto pOri_F_ and monitored the stability of the resulting pCdiBAI plasmid. In the absence of ampicillin selection, pCdiBAI was retained by 40–70% of *recA*^−^cells over 100 generations (Fig 2C). These results demonstrate that *cdi* genes are more effective stabilizing elements than previously characterized TA plasmid-addiction modules.

### Plasmid stabilization is an emergent property of the CDI mechanism

**Fig 3:**
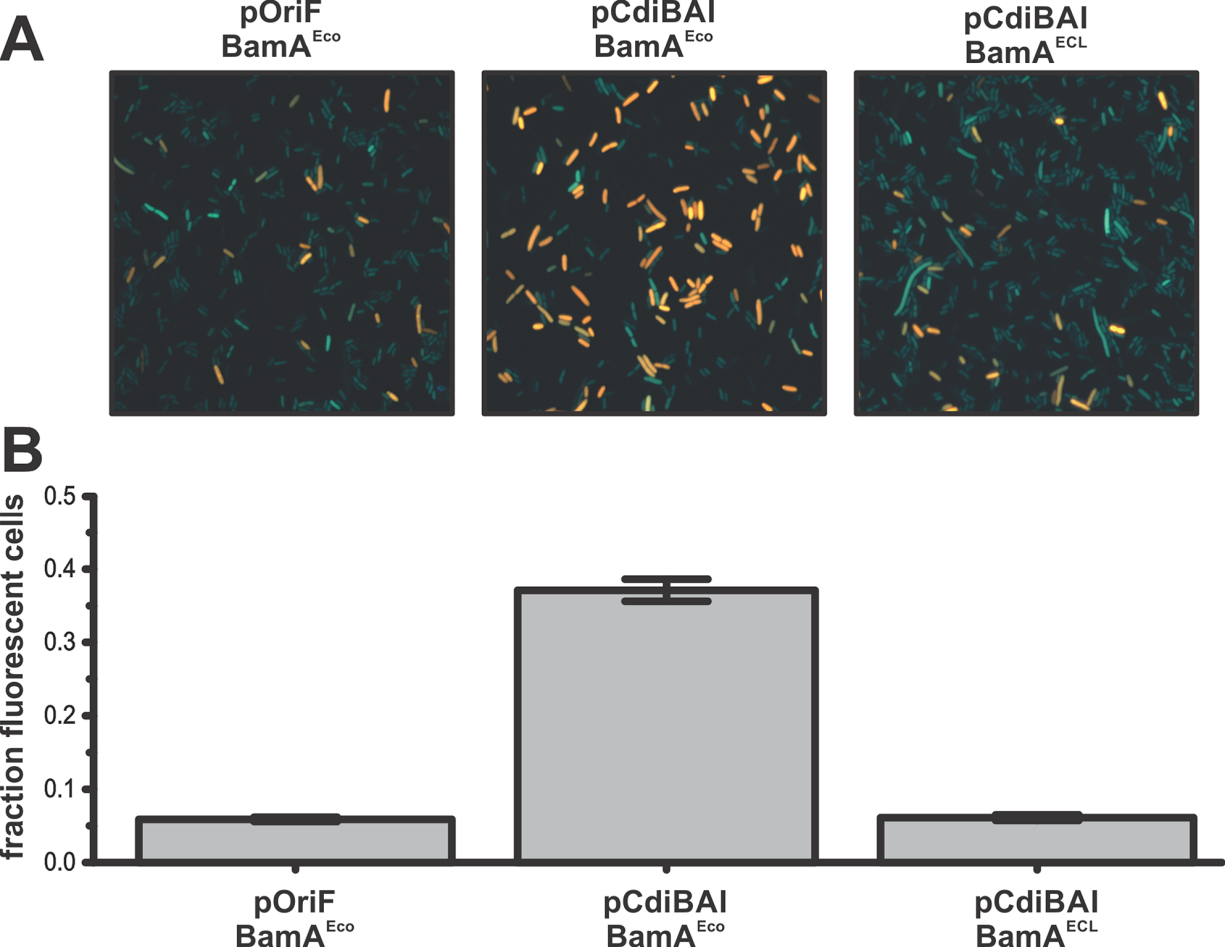
Ethidium bromide uptake analysis. **A**) *E*. *coli* EPI100 populations carrying the indicated mini-F derivatives were stained with EtBr and visualized by fluorescence microscopy. **B**) The fraction of EtBr-stained cells was quantified from the populations in panel A and presented as the average ± standard error of the mean.

**Fig 4:**
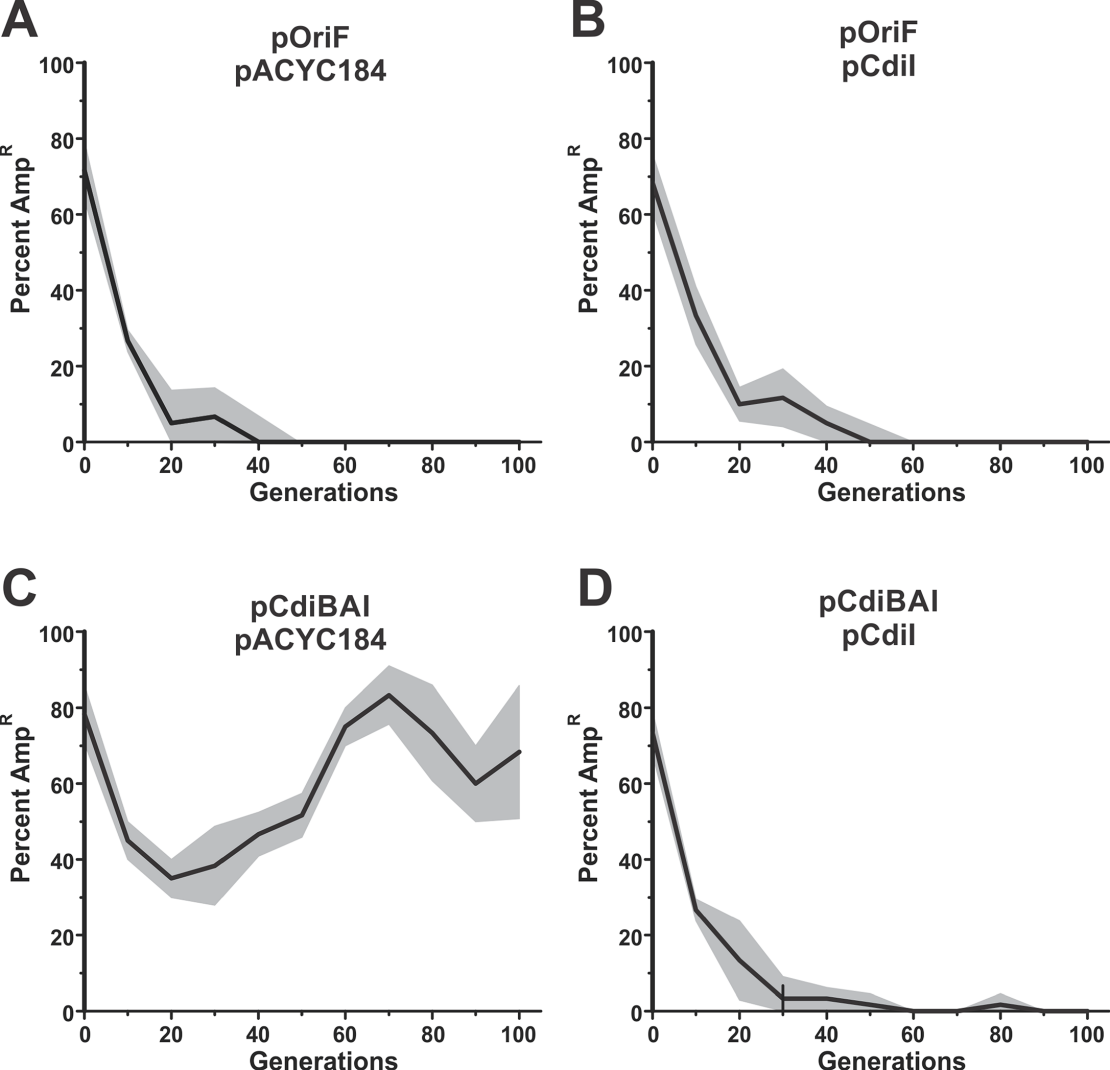
Plasmid stabilization reflects the selection for immunity to CDI. *E*. *coli* EPI100 populations harboring mini-F plasmid derivatives were passaged daily in broth culture for 100 generations. Randomly selected colonies were screened Amp^R^ after each passage to determine the percentage of cells that retain the plasmid. The average percentage of Amp^R^ colonies is plotted with the standard error indicated by grey shading. **A**) *E*. *coli* EP100 pOri_F_ pACYC184. **B**) *E*. *coli* EP100 pOri_F_ pCdiI. **C**) *E*. *coli* EP100 pCdiBAI pACYC184. **D**) *E*. *coli* EP100 pCdiBAI pCdiI.

In principle, *cdi* genes could stabilize the F episome through an auto-intoxication mechanism similar to TA-mediated plasmid addiction. Alternatively, genetic stability could be enforced through inter-cellular toxin delivery to inhibit plasmid-free cells. To differentiate between these two mechanisms, we examined pCdiBAI stability in *E*. *coli* cells that cannot exchange toxins with one another. CdiA^EC93^ uses the *E*. *coli* BamA protein as a cell-surface receptor to bind target bacteria [18], but does not recognize BamA from other bacterial species [37]. Therefore, we introduced pCdiBAI into *E*. *coli bamA*^ECL^ cells, which express heterologous BamA from *Enterobacter cloacae* and are completely resistant to CDI^EC93^ [37]. pCdiBAI was lost from *bamA*^ECL^ populations at the same rate as pOriF (Fig 2, compare panels A and D), suggesting that plasmid stabilization requires cell-to-cell delivery of CDI toxins. Thus, plasmid-free cells are likely eliminated from the population by toxin-deploying siblings that retain pCdiBAI. To test this model, we identified and enumerated CDI-intoxicated cells in populations that harbor pCdiBAI. CdiA^EC93^ delivers a pore-forming toxin that dissipates the proton-motive force in target bacteria [23]. Because the proton-motive force is required to export ethidium bromide (EtBr) dye, intoxicated bacteria become highly fluorescent when incubated with EtBr [38]. Fluorescence microscopy of populations that carry pCdiBAI revealed that 37% of the cells were stained with EtBr, compared to about 6% of cells in populations with pOri_F_ (Fig 3A and 3B). Moreover, *bamA*^ECL^ populations harboring pCdiBAI showed about the same percentage of fluorescent cells as the pOri_F_ population (Fig 3A and 3B). Thus, a significant proportion of cells in the pCdiBAI population show signs of CDI-mediated intoxication. Collectively, these data suggest that pCdiBAI is maintained in the population because it protects hosts from CDI-mediated cell killing. If this model is correct, then expression of the *cdiI* immunity gene from a second, unlinked locus should relieve the selective pressure to retain pCdiBAI. Therefore, we monitored F episome stability in cells that express *cdiI* from a pACYC184 multi-copy vector. The pACYC184 vector itself has no effect on pOriF stability (Fig 4A), nor did the pCdiI expression plasmid (Fig 4B). However, pCdiBAI was lost very rapidly from populations that also harbored pCdiI, similar to the pOriF/pCdiI control (Fig 4B and 4D). Collectively, these results indicate that pCdiBAI stability reflects the selective pressure to retain and express the *cdiI* immunity gene.

### Plasmid pCdiBAI becomes fixed in populations after long-term passage

**Fig 5:**
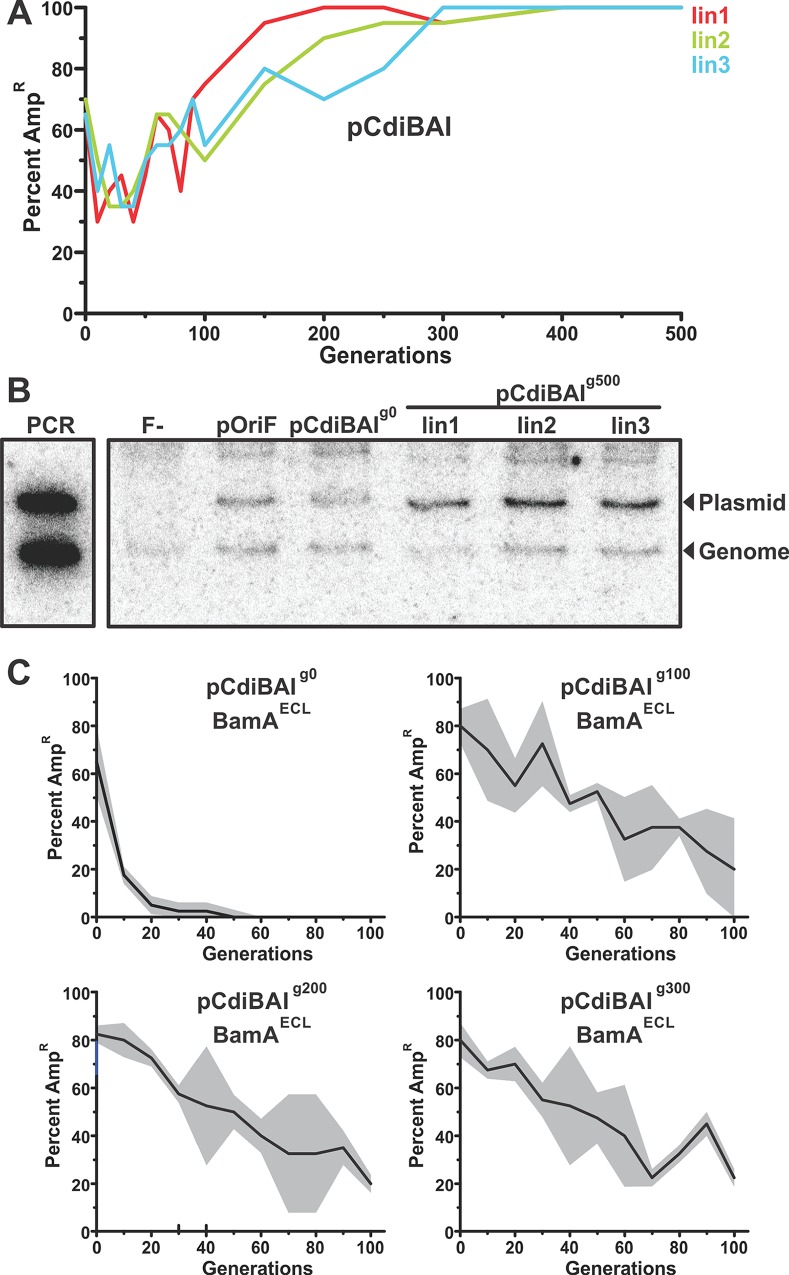
Plasmid pCdiBAI becomes fixed in populations after long-term passage. **A**) Three *E*. *coli* EPI100 pCdiBAI lineages were passaged for 500 cell generations, and the percentage of cells that carry plasmid pCdiBAI determined. **B**) Total DNA was isolated from the lineages shown in panel A after 500 generations (g500). DNA samples were digested with PstI and analyzed by Southern blot using radiolabeled probes specific for plasmid pCdiBAI and the *groL* chromosomal locus. Control samples isolated from cells lacking plasmid (F-), cells carrying pOri_F_ and unpassaged cells carrying pCdiBAI (pCdiBAI^g0^) were also analyzed. Molecular standards were generated by PCR of plasmid pCdiBAI and the genomic *groL* locus. **C**) Plasmid DNA was isolated from the lineages in panel A after 0 (g0), 100 (g100), 200 (g200) and 300 (g300) generations. The plasmids were transformed into *E*. *coli ΔbamA*::*cat* pZS21-BamA^ECL^ and the percentage of cells carrying plasmid pCdiBAI was monitored over 100 generations.

Plasmid pCdiBAI is maintained in about half the cell population over 100 generations. Strikingly, the percentage of cells carrying pCdiBAI increased upon further passage, with carriage approaching 100% in three independent lineages after 300 generations (Fig 5A). These results suggest that mutations in host cells or the plasmid allow the episome to be fixed in the population. Mutations in *repE* (*copA*), which encodes the F replication-initiator protein, are known to increase copy number per cell and represent a possible mechanism to increase plasmid stability [39, 40]. To explore this possibility, we used Southern blotting to estimate plasmid copy number relative to a chromosomal marker. Because the parental pCdiBAI plasmid (pCdiBAI^g0^) is carried by only half the cells in a population, we isolated DNA from ampicillin-supplemented cultures to ensure that all cells carry plasmid. Hybridization with probes to F-plasmid DNA and the chromosomal *groL* locus revealed that the copy number of pCdiBAI plasmids from generation 500 (g500) increased two- to four-fold compared to plasmid pOri_F_ and to the unpassaged (g0) pCdiBAI plasmid (Fig 5B). We also isolated pCdiBAI plasmids from each lineage after 100, 200 and 300 generations, then introduced the plasmids into *bamA*^ECL^ cells to assess stability in the absence of CDI selective pressure. Each "evolved" pCdiBAI plasmid was retained by a significant proportion of *bamA*^ECL^ cells after passage for 100 generations (Fig 5C). By contrast, the unpassaged pCdiBAI^g0^ plasmid was lost from the population within 50–60 generations (Fig 5C). These data indicate that the evolved plasmids are stabilized independent of CDI surveillance. We propose that prolonged passage allows the acquisition of mutations that increase copy number and fix the plasmid in the population.

### DNA stabilization by colicin/immunity protein systems

**Fig 6:**
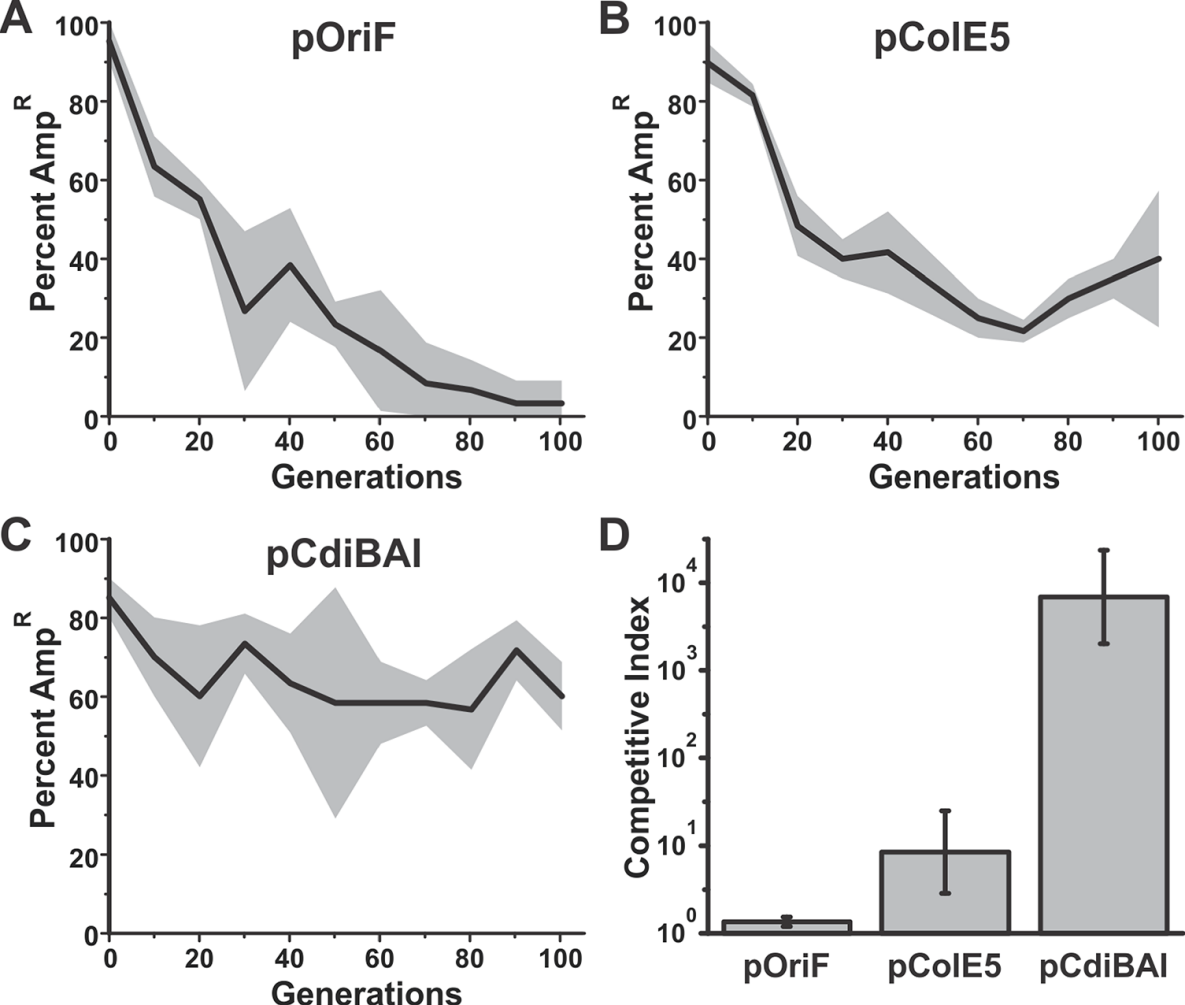
Colicin/immunity genes stabilize genetic elements. *E*. *coli* populations harboring mini-F plasmid derivatives were passaged daily in broth culture for 100 generations. Randomly selected colonies were screened for Amp^R^ after each passage to determine the percentage of cells that retain the plasmid. The average percentage of Amp^R^ colonies is plotted with the standard error indicated by grey shading. **A**) *E*. *coli* MG1655 pOri_F_. **B**) *E*. *coli* MG1655 pColE5. **C**) *E*. *coli* MG1655 pCdiBAI. **D**) Plasmid pColE5 provides a competitive advantage. *E*. *coli* MG1655 harboring the indicated mini-F derivatives were co-cultured at a 1:1 ratio with plasmid-free, Rif^R^ MG1655 cells. The competitive index was calculated as described in Methods. The average ± standard error is presented for three independent experiments.

Many *E*. *coli* isolates carry colicinogenic (Col) plasmids, which encode toxin/immunity systems that share some general features with CDI. Col^+^ strains release colicins, which are diffusible cytotoxic proteins that kill other *E*. *coli* strains. Colicins bind to specific receptors on *E*. *coli* and subsequently translocate their C-terminal toxin domains into the cell [41]. Col plasmids also encode specific immunity proteins that protect against colicin toxicity. These parallels with CDI suggest that colicin/immunity gene pairs should also stabilize genetic elements. To test this prediction, we placed the coding sequences for colicin E5 and its cognate ImE5 immunity protein onto pOri_F_ to generate plasmid pColE5. We compared the stabilities of plasmids pOri_F_ and pColE5 in *recA*^*+*^ cells (*E*. *coli* MG1655) since colicin expression is dependent on the SOS response to DNA damage and this pathway is defective in *recA* mutants. Plasmid pOri_F_ was somewhat more stable in recA^+^ cells compared to *recA*^**-^ cells, but the frequency of cells carrying pOri_F_ still declined rapidly over 100 generations (compare Figs 2A and 6A). Notably, plasmid pColE5 showed increased stability compared to pOri_F_ (Fig 6B), but was not stabilized to the same extent as plasmid pCdiBAI (Fig 6C). These data suggest that pColE5 provides a competitive advantage to cells, presumably because it encodes immunity protein to protect against colicin intoxication. We tested this hypothesis in competition co-cultures that were seeded with a 1:1 ratio of plasmid-free rifampicin-resistant (Rif^R^) cells with rifampicin-sensitive (Rif^S^) cells that harbor plasmid pColE5. After incubation for 24 h, we enumerated colony forming units for each population and found that Rif^S^ descendants of the pColE5 carrying population were 8.5 fold more numerous than Rif^R^ bacteria (Fig 6D). This competitive index is significantly greater than that of cells that carry pOriF, but not as great as the advantage provided by plasmid pCdiBAI (Fig 6D). Together, these results indicate that selective pressure to retain CDI and colicin immunity helps to stabilize genetic elements in a population.

### CDI systems are functional after horizontal transfer

**Fig 7:**
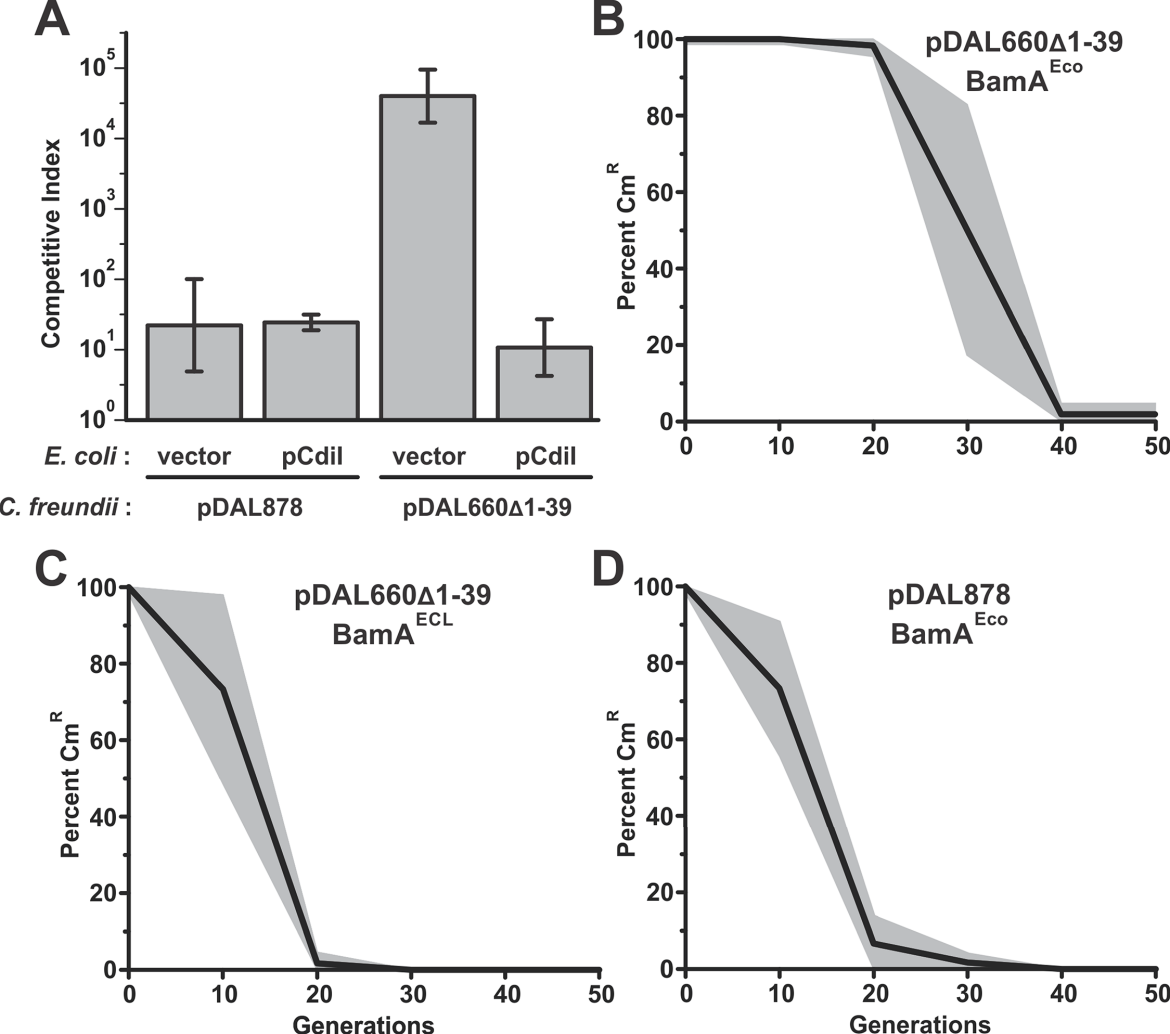
The *cdiBAI*^EC93^ genes are functional after horizontal transfer. **A)**. *C*. *freundii* cells harboring pDAL878-*cat* (CDI^–^) or pDAL660Δ1-39-*cat* (CDI^+^) were co-cultured with *E*. *coli* MG1655 target cells that carry pTrc99A (vector, Amp^R^) or pTrc99A::*cdiI*^EC93^ (pCdiI, Amp^R^). Competitive indices were calculated as the ratio of *C*. *freundii* to *E*. *coli* cells at 4 h divided by the initial ratio. The average ± standard error is presented for three independent experiments. **B to D).** *C*. *freundii* cells harboring pDAL660Δ1-39-*cat* (CDI^+^) or pDAL878-*cat* (CDI^–^) were passaged daily in broth culture for 50 generations. The cells also contained plasmids that express *bamA*^Eco^ or *bamA*^ECL^ where indicated. Randomly selected colonies were screened for Cm-resistance after each passage to determine the percentage of cells that retain the plasmid. The average percentage of Cm^R^ colonies is plotted with the standard error indicated by grey shading.

Purified two-partner secretion proteins are autonomous systems and have been shown to assemble and function properly in artificial membranes [42]. These findings, together with the observation that *cdi* genes are typically found on mobile genetic elements, suggest that CdiB/CdiA should function in many different Gram-negative species. To test this prediction, we asked whether the *cdiBAI* gene cluster from *E*. *coli* EC93 is functional when expressed in *Citrobacter freundii* ATCC 8090. We introduced plasmid pDAL660Δ1–39, which constitutively expresses *cdiBAI*^EC93^ [13], into *C*. *freundii* and asked whether the resulting cells deploy the CDI system. *C*. *freundii* cells carrying pDAL660Δ1–39 had a significant competitive growth advantage over *E*. *coli* target cells in competition co-culture (Fig 7A). This advantage was not observed with *C*. *freundii* cells that harbor plasmid pDAL878 (Fig 7A), which is a derivative of pDAL660Δ1–39 lacking the *cdiA-CT/cdiI* toxin/immunity coding sequences [28]. Moreover, *E*. *coli* targets were protected from inhibition when they express the *cdiI* immunity gene (Fig 7A). These data demonstrate that the CDI^EC93^ system is functional immediately after horizontal transfer into a different species.

We next examined plasmid stability in the *C*. *freundii* populations. Unlike the minimal F plasmid described above, pDAL878 and pDAL660Δ1–39 carry ColE1 replication origins and are maintained as multi-copy elements in *E*. *coli*. Nevertheless, we found that pDAL878 was lost from *C*. *freundii* populations within 30 generations when released from chloramphenicol selection (Fig 7D). We have previously shown that BamA from *C*. *freundii* is not a suitable receptor for CdiA^EC93^ [37], suggesting that pDAL660Δ1–39 should also be lost rapidly from *C*. *freundii* populations. Therefore, we assessed pDAL660Δ1–39 stability in *C*. *freundii* cells that ectopically express either *bamA*^Eco^ or *bamA*^ECL^ from a second plasmid. As predicted, pDAL660Δ1–39 was retained in *bamA*^Eco^ expressing populations (Fig 7B) longer than in *bamA*^ECL^ populations (Fig 7C), but the element was still lost after 40 generations. These results suggest that the stabilizing effect of CDI systems is less efficient in unrelated host cells, due in part to the specificity of CdiA-receptor interactions.

## Discussion

The results presented here show that CDI exerts a powerful selective pressure to retain genetic elements. The *cdi* genes from *E*. *coli* EC93 significantly stabilize a minimal F plasmid, allowing cells to retain this intrinsically unstable episome for more than 100 generations. However, the system has no effect in populations that lack receptors for CdiA^EC93^, indicating that toxin exchange between cells enforces plasmid retention. In accord with this model, a significant proportion of cells in the population show signs of CDI-mediated intoxication, suggesting that they have lost immunity because they no longer harbor the *cdi* plasmid. Moreover, the *cdi* plasmid is rapidly lost from cells that carry a second copy of the *cdiI* immunity gene on a separate plasmid. Collectively, these results indicate that CDI stabilizes genetic elements through a surveillance mechanism. Cells that retain the *cdi* plasmid remain immune, but plasmid-free cells lose immunity and are eliminated by their CDI^+^ siblings. This mechanism differs from the toxin-induced cell suicide strategy used by type II TA systems. Post-segregational killing relies on the rapid degradation of antitoxin to liberate cognate toxin in plasmid-free cells. Results presented here and elsewhere show that the *ccdAB* TA system of F is not wholly effective in killing plasmid-free daughter cells [9, 36, 43]. In principle, cells could escape post-segregational killing if the CcdA antitoxin is not degraded in a timely manner. Additionally, escape may reflect recovery from CcdB intoxication. CcdB poisons DNA gyrase and induces breaks in the genome [44–46]. This activity blocks cell division and is potentially lethal. However, the lesions generated through CcdB activity also induce the SOS response, which could conceivably repair the DNA damage and promote survival. *E*. *coli* encodes an endogenous gyrase inhibitor (SbmC/GyrI) that is induced by the SOS response [47]. GyrI ameliorates the toxic effects of CcdB and has been proposed to defend against post-segregational killing [48]. Regardless of mechanism, cells that escape post-segregational killing are free to replicate with no further consequence. By contrast, individuals that lose CDI immunity are recurrently challenged by other cells in the population. This continual selection presumably provides a greater pressure to retain *cdi* associated genetic elements.

Community-based selection appears to be a general property of toxin/immunity competition systems. Our results show that the minimal F episome is stabilized when it expresses ColE5 toxin and its cognate ImE5 immunity protein. These results are generally consistent with previous studies showing that colicinogenic (Col^+^) bacteria have a competitive advantage over plasmid-free cells [49–51]. This growth advantage is dependent on the initial frequency of Col^+^ bacteria in the population. The serial passage experiments described by Chao & Levin are most similar to our approach, and they reported Col^+^ bacteria rapidly dominate co-cultures when present at an initial frequency of at least 10^−2^ [50]. At lower initial frequencies, Col^+^ bacteria are outcompeted and eventually are lost from the population. The disadvantage at low initial frequencies is presumably due to slower growth rates for Col^+^ bacteria and the fact that Col^+^ cells must undergo lysis and die to release colicins into the environment. However, Col^+^ bacteria enjoy a much greater advantage in a structured soft-agar environment, where they dominate co-cultures from initial frequencies as low as 10^−6^ [50]. This latter phenomenon reflects the ability of colicins to diffuse through the medium and kill at a distance. A zone of inhibition surrounds each Col^+^ colony, allowing colicinogenic cells to monopolize resources within the sphere of influence. We predict that other inter-bacterial competition systems likely exert the same selective pressures. For example, bacterial type VI secretion systems have been shown to be potent inter-bacterial competition systems, capable of killing many different Gram-negative species [52–54]. Like CDI systems and colicins, type VI secretion systems are encoded by mobile genetic elements [55–58], and different strains of a given species typically deploy a distinct complement of toxic effector proteins [59]. Together, these observations suggest that competitive systems reflect a general strategy to reduce genetic heterogeneity. This is accomplished through growth inhibition of competing bacteria and selective inhibition of sibling segregants that have lost toxin immunity genes.

Metagenomic analyses show that *cdi* genes are found on genomic islands in several species including *Bartonella henselae* [60], *Burkholderia pseudomallei* [61, 62], *Histophilus somni* [63] and *Neisseria meningitidis* [64]. In addition, CDI systems are also commonly encoded on large low-copy plasmids. *Cronobacter* species carry a virulence plasmid that encodes predicted CDI systems [55, 65], and Shiga toxin producing O104 and O113 strains of *E*. *coli* carry their *cdi* genes on a plasmid [66, 67]. In fact, the *E*. *coli* EC93 *cdi* genes used in this study reside on a large plasmid (manuscript in preparation). Together with our findings, these observations suggest that genetic stabilization is a physiologically relevant function of *cdi* genes. Hacker and colleagues have isolated UPEC 536 cells lacking the PAI II_536_ island due to excision events, which generate non-replicating circular intermediates [3, 68]. However we found that despite PAI II_536_ excision in UPEC 536 strains, long-term passaging failed to yield a significant population of cells lacking the island. These results are consistent with similar studies showing that genomic islands are stable through multiple generations [29, 30]. We note that PAI II_536_ carries a large number of genes that may affect retention, including the *yeeUV* toxin-antitoxin module. Therefore, the stability of PAI II_536_ and other genomic islands is likely determined by the collective action of its beneficial genes, gene loss events, and selective inhibition of segregants.

*E*. *coli cdi* gene clusters show evidence of horizontal transfer at at least two levels. First, *cdi* genes are clearly located on large genomic islands that are integrated into the chromosome at the 3´-ends of tRNA genes. These islands are distributed sporadically across *E*. *coli* isolates and are absent from the K-12 derivatives commonly used in molecular biology laboratories. Second, the *cdi* genes within a given family of genomic islands often encode different toxin/immunity protein pairs, indicating that *cdiA-CT/cdiI* sequences can be replaced through recombination. Moreover, the regions downstream of the *cdiI* immunity gene vary considerably within each island family and contain fragmented *cdiA-CT/cdiI* gene pairs in the same transcriptional orientation as the *cdiBAI* cluster. We previously identified these modules as "orphan" toxin/immunity gene pairs, because they appear to be the displaced 3´-ends of *cdi* clusters [28]. Orphan coding regions also typically contain predicted integrase and transposase genes, suggestive of recent horizontal transfer into the island. We propose two models to explain the existence and physiological function of orphan modules. Orphan toxin/immunity genes could represent a "fossil" record of past HGT events in which ancestral *cdiA-CT/cdiI* sequences are preserved. In this model, newly acquired *cdiA-CT/cdiI* modules integrate into *cdiA*, thereby displacing the original toxin/immunity coding sequences downstream. Reiteration of this process could generate the tandem *cdiA-CT/cdiI* arrays that trail many *cdi* gene clusters [28]. Presumably, these orphan modules are retained for their immunity function, thereby preventing growth inhibition by neighboring parental cells. Alternatively, orphan pairs could function as a repository of “silent” toxin/immunity genes held in reserve for future deployment. Many orphan *cdiA-CT* fragments retain conserved sequences that are homologous to the upstream *cdiA* gene, and therefore homologous recombination could fuse orphan *cdiA-CT/cdiI* modules to *cdiA*. Cells undergoing such recombination would deploy a new CdiA-CT toxin, which could provide a competitive advantage under some circumstances. However, this recombination event would also delete the original *cdiI* immunity gene, leaving recombinants susceptible to the toxin delivered by parental cells. Alternatively, if the *cdi* locus was duplicated prior to homologous recombination, then a copy of the *cdiI* gene would remain, thereby enabling the recombinant to deploy a new toxin *and* retain immunity to the parental system. Chromosomal duplications are common in bacteria, and a given population will contain duplications of essentially every region of the genome [69]. Recent experiments with "evolved" *Salmonella* Typhimurium LT2 support the duplication-recombination model for orphan toxin utilization [70]. *S*. Typhimurium does not carry *cdi* genes, but possesses an analogous *rhs* (rearrangement hotspot) gene cluster that encodes a large peptide-repeat protein with a C-terminal toxin domain together with a cognate RhsI immunity protein. This locus also contains an orphan *rhs-CT/rhsI* module, which can recombine with the upstream *rhs* gene to deploy the orphan toxin. In this manner, a subpopulation of evolved cells inhibits ancestral cells with orphan toxin. Notably, reversion of this recombination can restore the ancestral *rhs* locus. Therefore, evolved orphan expressing cells is presumably a dynamic process in which orphan-expressing cells appear and disappear from the population. This model predicts that *rhs* (or *cdi*) recombinants do not become fixed in the population, but nevertheless these cells exert a selective pressure, perhaps explaining why orphan toxin-immunity gene pairs are retained.

HGT occurs most efficiently between closely related species [4]. For example, transfer between different haloarchaea decreases exponentially with evolutionary distance [71]. Several factors account for this bias. Some genetic competence systems preferentially take up DNA from related bacteria, and restriction endonucleases destroy DNAs that lack "self" modification patterns. Moreover, integration into the genome via homologous recombination requires relatively long tracts of sequence identity [72]. CDI surveillance should also preferentially stabilize genetic elements from closely related bacteria, because this mechanism requires recognition of specific receptors. Accordingly, the *E*. *coli* EC93 *cdi* genes do not stabilize plasmid in *C*. *freundii* populations, because these cells lack the appropriate CdiA receptor. Though surveillance is only possible when host cells express CdiA receptors, newly acquired *cdi* genes could provide other advantages. For example, CdiA^EC93^ contains a homotypic interaction domain that promotes BamA-independent auto-aggregation and biofilm formation [73]. Biofilms protect against a variety of environmental insults including oxidative stress, antibiotics, immune responses and predation. Unfortunately, the *C*. *freundii* cells used in this study adhere strongly to one another, making it difficult to determine whether CdiA expression affects auto-aggregation. Nevertheless, the self-adhesive properties of CdiA are potentially beneficial and could help to stabilize horizontally transferred *cdi* genes in the absence of surveillance.

## Materials and Methods

### Plasmid constructions

All mini-F plasmids are derivatives of pML31 [74]. The *bla* gene was amplified from pWEB-TNC using primers **Amp-Hind-for** (5´—TTT AAG CTT GCG GCC GCT TGA AGA CGA AAG GGC CTC G) and **Amp-Eco-rev** (5´—TTT GAA TTC TTG GTC TGA CAG TTA CCA ATG C). The resulting PCR product was ligated to the 8.7 kbp EcoRI/HindIII fragment of plasmid pML31 to produce pMiniF-Amp. pMiniF-Amp was digested with EcoRI and MfeI and re-ligated to remove the *sopABC* partitioning locus and produce plasmid pCcdAB. pCcdAB was amplified with **Amp-Hind-for** and **OriF-Hind-rev** (5´—TTT GGA TCC GCA AGC TTG GAA TAT AAA TGT CAG G) and the resulting product digested with HindIII and re-ligated to produce pOri_F_. The *E*. *coli* EC93 *cdiBAI* gene cluster was amplified using oligonucleotides **cdiB-Not-for** (5´—TTT GCG GCC GCA ACA CCT GAA CTG GAA CTT GTG) and **cdiI-Not-rev** (5´—TTT GCG GCC GCT ATT TTC TGT CTA AGA TAC TAA GGC C) and cloned into pOri_F_ using Not I digestion to produce pCdiBAI. The *col-imm-lys* gene cluster was amplified from plasmid ColE5-099 using primers **colE5-Not-for** (5´—TTT GCG GCC GCA CCT GAT GCA GTT C) and **colE5-Not-rev** (5´—TTT GCG GCC GCT CAA AAC GAC AAG TTT TCT CC) and cloned into pOri_F_ using the NotI restriction site to produce pColE5. Plasmid pDAL660Δ1–39 expresses the *E*. *coli* EC93 *cdiBAI* gene cluster constitutively and contains a ColE1 origin of replication [13].

### Cell passage and plasmid stability

Overnight cultures of *E*. *coli* EPI100 (*recA*^*–*^) or MG1655 (*recA*^+^) harboring mini-F plasmid derivatives were grown from frozen stocks in lysogeny broth (LB) supplemented with 100 μg/mL ampicillin (Amp). The following day, the cultures were diluted 2^−10^ in 2 mL fresh LB without Amp, and incubated overnight (corresponding to ~10 generations) at 37°C on roller drum. This process was repeated iteratively for three independent population lineages for ≥100 generations. At every passage, aliquots of overnight cultures were diluted and plated on LB-agar. 20 colonies were selected randomly and screened for ampicillin resistance (Amp^R^) on LB-agar supplemented with 100 μg/mL Amp to determine the fraction of cells carrying plasmid.

### Ethidium bromide staining

*E*. *coli* harboring mini-F plasmids were grown overnight in LB medium supplemented 100 μg/mL Amp. The following day, cultures were diluted 1:100 into 2 mL of fresh LB medium without Amp and grown to OD_600_ ~ 1.0 at 37°C. Cells were harvested by centrifugation at 6,000 *g* and washed once in pre-warmed (37°C) 1 M9 salts. Cells were then resuspended in pre-warmed 1 M9 salts supplemented with 25 μM ethidium bromide (EtBr). After incubation for 2 to 5 min, cells were harvested by centrifugation and washed twice with pre-warmed 1 M9 salts. Cells were mounted on agarose pads for imaging by fluorescence microscopy. EtBr staining was scored by counting fluorescent cells and total cells (fluorescent + non-fluorescent) within images of randomly selected fields using fluorescence microscopy [19].

### Southern blot analysis

Total DNA was isolated as described previously [70]. DNA (10 μg) was digested with PstI and resolved on 6% polyacrylamide gels buffered with Tris-borate-EDTA. Gel-mobility standards were generated by PCR using oligonucleotide pairs **MiniF-std-for** (5´—CAG TCA TGG TAC CGG CAG) and **MiniF-std-rev** (5´—CAG CTG GCT GAC GTA CC); and **groL-std-for** (5´—CAG GAC GAA CTG GAC GTG) and **groL-std-rev** (5´—CAG CAT AGC TTT ACG ACG ATC G). Gels were electroblotted onto nylon membrane and the blots hybridized with radiolabeled oligonucleotides **MiniF-probe** (5´—CAG CAG ATA TTT TGG CAG TTC A) and **groL-probe** (5´—GAA GTA AGG AGA CAG GTA GCC). Autoradiograms were visualized using a Bio-Rad PMI phosphorimager and Quantity One software.

### Co-culture conditions

*E*. *coli* MG1655 "inhibitor" strains harboring pOriF, pCdiBAI or pColE5 were mixed at a 1:1 ratio with rifampicin-resistant (Rif^R^) MG1655 target cells in LB media without antibiotics at a final OD_600_ of 0.1. Co-culture aliquots were plated onto LB-agar and LB-agar supplemented with 200 μg/mL Rif to enumerate initial cell counts as colony-forming units (CFU) per mL. Inhibitor cell counts were determined by subtracting Rif^R^ CFU from total CFU on antibiotic-free agar. Co-cultures were incubated for 24 h at 37°C with agitation, and strain counts determined by plating on non-selective and Rif-supplemented agar. For experiments in which antibiotic resistant plasmids were present (Fig 7), cells were cultured overnight in LB medium containing the appropriate antibiotic, diluted 1:100 in antibiotic free LB medium prior to competition mixing. The competitive index was calculated as the ratio of inhibitor cells to target cells at 24 h divided by the initial inhibitor to target cell ratio. Reported competitive indices represent the average ± standard error for three independent experiments.

Plasmids pDAL878-*cat* and pDAL660Δ1-39-*cat* were introduced into *Citrobacter freundii* ATCC 8090 cells by electroporation and transformants were selected on LB-agar supplemented with 33 μg/mL chloramphenicol (Cm). pDAL660Δ1-39-*cat* constitutively expresses the *E*. *coli* EC93 *cdiBAI* genes, and pDAL878-*cat* is a derivative in which the *cdiA-CT/cdiI* toxin/immunity sequences have been deleted [28]. *C*. *freundii* inhibitors that carry pDAL660Δ1-39-*cat* were co-cultured at a 10:1 ratio with *E*. *coli* MG1655 *ara*::*spec* target cells that harbor plasmid pTrc99a or pTrc99a::*cdiI*^EC93^ [38]. Initial cell counts were determined by plating the co-culture on LB-agar supplemented with 33 μg/mL Cm to enumerate *C*. *freundii* inhibitors and 50 μg/mL spectinomycin to enumerate *E*. *coli* targets. Co-cultures were incubated at 37°C with agitation for 4 h, then plated again on selective media to determine inhibitor and target cell CFU/mL. The competitive index was calculated as the ratio of inhibitor cells to target cells at 24 h divided by the initial inhibitor to target cell ratio. Reported competitive indices represent the average ± standard error for three independent experiments.
